# A Unique Case of Fatal Coinfection Caused by *Leptospira* spp. and *Hepatozoon canis* in a Red Fox Cub (*Vulpes vulpes*)

**DOI:** 10.3390/pathogens11010011

**Published:** 2021-12-22

**Authors:** Amer Alić, Jovana Šupić, Teufik Goletić, Emina Rešidbegović, Ismar Lutvikadić, Adnan Hodžić

**Affiliations:** 1 Department of Pathology, Faculty of Veterinary Medicine, University of Sarajevo, Zmaja od Bosne 90, 71000 Sarajevo, Bosnia and Herzegovina; jovana.supic@vfs.unsa.ba; 2 Department of Avian Diseases and Management, Faculty of Veterinary Medicine, University of Sarajevo, Zmaja od Bosne 90, 71000 Sarajevo, Bosnia and Herzegovina; teufik.goletic@vfs.unsa.ba (T.G.); emina.residbegovic@vfs.unsa.ba (E.R.); 3 Department of Surgery, Faculty of Veterinary Medicine, University of Sarajevo, Zmaja od Bosne 90, 71000 Sarajevo, Bosnia and Herzegovina; Ismar.lutvikadic@vfs.unsa.ba; 4 Department of Pathobiology, Institute of Parasitology, University of Veterinary Medicine Vienna, Veterinaerplatz 1, 1210 Vienna, Austria; Adnan.Hodzic@vetmeduni.ac.at

**Keywords:** Bosnia and Herzegovina, fox cub, *Hepatozoon canis*, *Leptospira*, pathology, *Vulpes vulpes*

## Abstract

Red foxes are the most abundant wild carnivore species in Europe commonly exposed to pathogenic *Leptospira* and *Hepatozoon canis*. Despite high seroprevalence, the clinical disease caused by these pathogens in red foxes has never been reported. Herein, we report the first-ever case of a fatal *Leptospira* spp. and *H. canis* coinfection in a two-month-old red fox cub with acute haemolytic anaemia, mild bronchopneumonia, intraalveolar haemorrhage, and tubulonephrosis. The presence of pathogenic *Leptospira* spp. DNA was detected in the kidney and lung tissues of the infected animal. In contrast to our previous knowledge, we believe that such fatal cases due to concomitant infection by *Leptospira* spp. and *H. canis*, especially in young animals, may commonly occur in nature. However, further studies are required to identify other factors that possibly contribute to the severity and the pathogenic effect of *Leptospira* spp. and *H. canis* infections in red foxes.

## 1. Introduction

Red foxes (*Vulpes vulpes*) are the most common and widespread wild carnivores in Europe, and they are well-recognised hosts for many pathogens shared between wild and domestic animals and humans as well. Many of these pathogenic agents cause severe illnesses in domestic animals and may represent a significant threat to public health [1,2,3].

The exposure of red foxes to re-emerging pathogens like pathogenic *Leptospira* are frequently reported. The seroprevalence of leptospirosis in red foxes varies among regions and can reach up to 47% [1,4,5,6,7]. The variations are probably the result of differences in targeted serovars and their regional circulation and endemicity [1].

Another commonly detected pathogen in red foxes is *Hepatozoon canis* (Adeleorina, Hepatozoidae), a common tick-transmitted pathogen affecting dogs and other canids worldwide. The red fox is considered as a potential reservoir host for *H. canis* in Europe, and the prevalence can reach up to 95% in some countries [8,9,10,11,12,13,14,15,16]. Despite the high prevalence observed by molecular methods, histopathology revealed a low number of *H. canis* meront stages with no apparent tissue response in the infected animals [10].

Pathogenic *Leptospira* and *H. canis* often cause clinical disease in dogs affecting many organ systems, with a potentially lethal outcome. Lesions induced by *Leptospira* spp. are most commonly localised in the kidneys and liver, but many other organs can also be affected [17,18]. In addition, a new Leptospiral Pulmonary Haemorrhagic Syndrome (LPHS) has recently been recognised in dogs [18,19]. Hence, the clinical manifestation of leptospirosis ranges from mild to severe and is related to the organ system involved [17,18]. The infection in dogs with *H. canis* ranges from a common subclinical to a life-threatening clinical condition characterised by a high parasite load, lethargy, fever, cachexia, anaemia, and leucocytosis [20,21,22]. At necropsy, hepatitis, interstitial pneumonia, and glomerulonephritis associated with the development of numerous *H. canis* meronts in the corresponding organs and, also, in the bone marrow, spleen, and lymph nodes are the most common findings in dogs with severe infections [21,23].

In contrast to dogs, despite a high seroprevalence, leptospirosis and hepatozoonosis seem to be harmless diseases in foxes, as there have been no clinical cases of infections documented so far [10,24]. Moreover, the association of pathogenic *Leptospira* with interstitial nephritis commonly observed in red foxes [25] has not been confirmed. Concurrent infections with other pathogens, especially in young animals, which are stressful and immunosuppressive situations, can be considered as contributing factors involved in the intensification of *H. canis* infection as a life-threatening condition [20,21]. In that sense, clinical illness and fatal cases due to *Leptospira* spp. and *H. canis* infection in commonly exposed free-ranging red foxes may likely occur. In the present work, we describe the first known case of a fatal coinfection caused by *Leptospira* spp. and *H. canis* in a juvenile red fox.

## 2. Case Report

In May 2019, a two-month-old female red fox cub was brought to the Clinics of the Faculty of Veterinary Medicine in Sarajevo (Bosnia and Herzegovina) with a suspected fracture of the right front limb. The cub was found on a hunting ground near Sarajevo. At clinical examination, she showed poor body condition, dehydration, lethargy, and hypothermia (35.9 °C). Seromucous nasal discharge, jaundice, expiratory stridor, and auscultation abnormalities in the thorax were also observed. The blood glucose level was 2.0 mmol/L, and the urine strip test showed no changes. The rapid canine distemper and canine adenovirus (CDV/CAV) antigen test (BioNote Inc., Hwaseong, Korea) was negative, while coprological examination revealed low numbers of *Cystoisospora* spp. oocysts and *Toxocara canis* eggs. The radiologic examination confirmed the fracture of the midshaft of the right humerus. The cub refused the food and was found dead the following day without any previous treatment. The carcass was subjected to necropsy.

**Figure 1:**
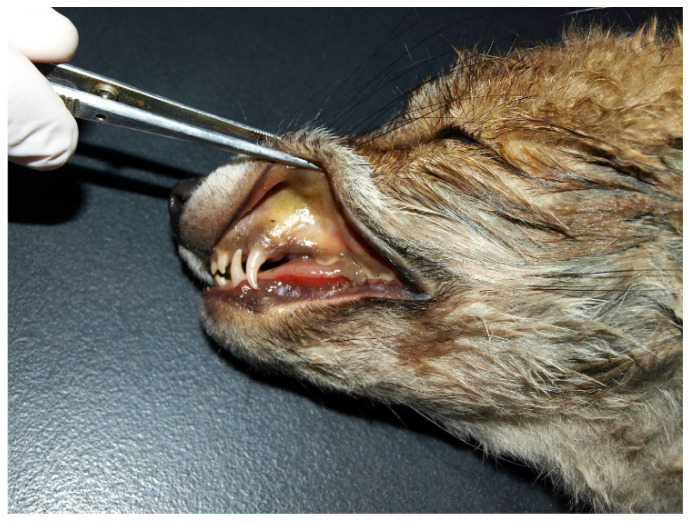
Head of the fox cub infected with *Hepatozoon canis* showing a diffuse brown–yellow discoloration of the oral mucosa (jaundice).

**Figure 2:**
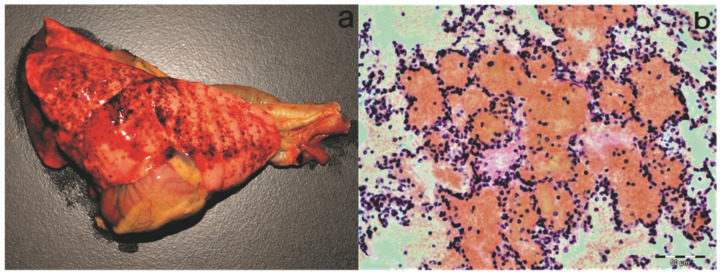
Macro- and microscopic changes in the lungs of the infected fox cub. (**a**) Multifocal petechial and ecchymotic haemorrhages are visible in all the lung lobes. (**b**) Histopathology of the lungs revealed multifocal blood-filled alveoli. A single alveolar space contains light eosinophilic fibrinous exudate. H&E staining. Scale bar: 50 µm.

At necropsy, all the visible mucous membranes (Figure 1), subcutis, fasciae, and visceral membranes were diffusely yellow. The spleen and all superficial lymph nodes, as well as mesenteric lymph nodes, were enlarged. The liver was slightly enlarged and brown to dark red. The gastrointestinal tract was empty, and no parasites were found. The lungs were firm wet and oedematous, with the right lung lobe discoloured red to dark red. Disseminated petechial and ecchymotic haemorrhages were present in all the lobes (Figure 2a). The tracheal mucosa was tinged red, and there was slight distension of the oesophagus. A small amount of light red serous liquid was present in the pericardial sac. Multifocal petechial and ecchymotic haemorrhages were observed on the pericardium, epicardium, and endocardium of the papillary muscles of the left ventricle. Nasal conchae were oedematous and diffusely discoloured orange–yellow, with the air passages filled with yellowish serous to mucous material. In the midshaft of the right humerus, there was a transverse fracture surrounded with haemorrhage and oedema. Samples of all the organs were collected for histopathology. Cytology of the blood smear stained with May–Grünwald–Giemsa showed severe anaemia with bright red to clear red blood cells. However, no blood parasites or parasitic stages were found in the smears.

**Figure 3:**
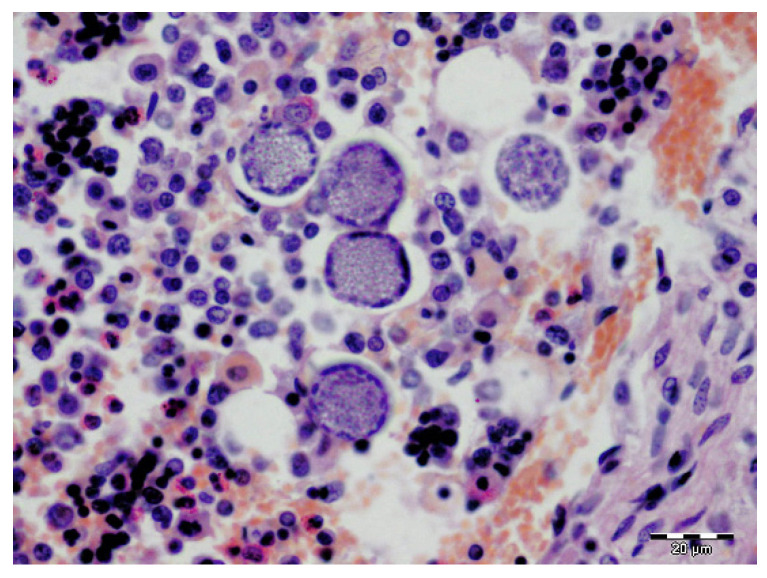
A cluster of five developing meronts of *Hepatozoon canis* in the bone marrow. H&E staining. Scale bar: 20 µm.

**Figure 4:**
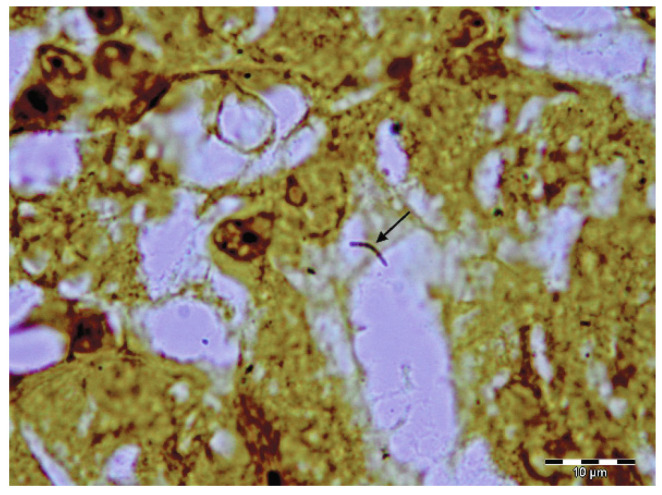
A single thin *Leptospira* bacterium in the kidney tubule of the infected fox cub (arrow). Warthin–Starry staining. Scale bar: 10 µm.

Histopathology revealed multifocal to coalescing areas of alveoli filled with red blood cells (haemorrhage) and, less frequently, light eosinophilic fibrinous exudate (Figure 2b) throughout the lung parenchyma of all the lobes. In the apical and cardiac lobes, a small number of a mixed population of eosinophils, alveolar macrophages, lymphocytes, and neutrophils was seen in the alveoli. Mild-to-moderate thickening of the alveolar septa observed throughout the parenchyma was most prominent in the diaphragmatic lung lobes. Mild-to-moderate perivascular oedema was also evident. The kidney tubules were slightly swollen with hypereosinophilic cytoplasm, and a few lymphocytes were present in the surrounding interstitium. The lymph nodes and spleen were moderately oedematous, and there was mild follicular hyperplasia observed in the spleen. Numerous early developing and mature meronts of *Hepatozoon* spp. were detected in the bone marrow (Figure 3), spleen, lymph nodes, and diaphragmatic lung lobes. The number of meronts in the bone marrow and spleen was classified as very high, according to the previously published grading system [26], while the lymph nodes and lungs were mildly parasitised. A special histochemical silver stain (Warthin–Starry) revealed a few small and thin bacteria in the sections of the kidney (Figure 4) but not in the lung tissue.

The presence of pathogenic *Leptospira* DNA was confirmed in the lungs and kidneys of the infected cub by a real-time PCR assay targeting the *secY* gene [27]. In addition, DNA extracts from the blood, bone marrow, spleen, and popliteal lymph nodes were subjected to PCR for the molecular detection of blood-borne pathogens (*Babesia, Hepatozoon, Leishmania*, Anaplasmataceae, *Rickettsia, Bartonella*, and hemotropic mycoplasmas) following the procedures described elsewhere [28]. Among the targeted pathogens, only *H. canis* was detected in the tissues, and the species identity was confirmed by bidirectional sequencing of the 18S rDNA gene (100% identity to, e.g., MK107808).

## 3. Discussion

To the best of our knowledge, this is the first fatal case of *Leptospira* spp. and *H. canis* coinfection in red foxes described so far. Even though *Leptospira* spp. and *H. canis* are highly prevalent throughout Europe [1,4,5,6,7,8,9,10,11,12,13,14,15,16], clinical cases caused by these pathogens have never been documented in red foxes before. The data on the pathology caused by pathogenic *Leptospira* and *H. canis* in carnivores were all derived from dogs and cats with severe and life-threatening illnesses [18,21].

Anaemia was the most prominent clinical sign in the current case, which is also one of the most commonly observed findings in dogs affected by leptospirosis [18] and hepatozoonosis [20,21]. Diffuse brown–yellow discoloration of various tissues (jaundice) in the infected cub suggests acute haemolytic anaemia. Anaemia in the present case is most probably the result of blood loss through respiratory haemorrhage or haemolysis due to leptospiral toxins [18].

The finding of multifocal mild purulent pneumonia accompanied with fibrinous exudate and intraalveolar haemorrhage in the present case is also consistent with that observed in dogs naturally infected by pathogenic *Leptospira* [18]. The multiple intraalveolar haemorrhagic foci observed in the lung parenchyma could be associated with LPHS, a severe and often lethal manifestation of acute leptospirosis [18,19]. Other lesions that can be present in dogs with LPHS, such as intraalveolar oedema, necrosis of the alveolar septa, hyaline membranes, and microvascular fibrin or hyaline thrombi were not seen in the lungs of the infected cub. These are characteristics of acute respiratory distress syndrome (ARDS), which could be initiated with various triggers [29].

Tubulonephrosis observed in the present case is also a consistent finding of leptospirosis in dogs [18]. However, the special histochemical silver staining (Warthin–Starry) for spirochetes showed a meagre number of the bacteria in the kidney, but not in the lung tissue, sections. The failure to visualise *Leptospira* in the lung is in congruence with the observation that they do not colocalise with pulmonary lesions (LPHS) in canine leptospirosis [18]. Furthermore, the inflammatory response in the form of lymphoplasmacytic cell infiltrates and interstitial perivascular haemorrhage commonly observed in acute canine leptospirosis [18] were absent in the examined fox kidneys.

The overall prevalence of *H. canis* in red foxes in Bosnia and Herzegovina reaches 38.6% [14], and the presence of its tick vector *Rhipicephalus sanguineus* sensu lato has also been recorded in the country [30]. However, the cub presented here could also have acquired the pathogen through vertical (transplacental) transmission from the infected vixen, as previously suggested [31]. In our large histopathology datasets, which include over 1500 red fox samples, such a high intensity of *H. canis* infection, as observed in this case, has never been documented. An extremely high number of *H. canis* meronts found in the bone marrow and spleen of the infected cub indicates a massive infection, which likely correlates with the severe clinical presentation. This observation is in line with previous studies showing that these two organs are the most heavily parasitised tissues in red foxes [10,24]. Coinfections, stressful and immunosuppressive situations, are considered contributing factors involved in the intensification of *H. canis* infection as a life-threatening condition [20,21]. This might be the case in the fox cub presented here, as it was found to be coinfected by *Leptospira* spp., which could potentially contribute to the severity of the *H. canis* infection. In addition, trauma due to the limb fracture and age of the animal (immature immune response) may have facilitated the overgrowth of detected pathogens and the subsequent increase in the severity of the infection.

Other parasitic, bacterial, and viral agents that could cause similar clinical conditions were ruled out by PCR, the rapid CDV/CAV antigen test, and pathology. For instance, canine infectious hepatitis caused by canine adenovirus type 1 (CAV-1) has been associated with several fatal outbreaks in juvenile red foxes [32,33]. However, based on the negative results obtained by the highly sensitive (>98%) antigen test and the absence of the characteristic lesions with inclusion bodies in the liver and kidney tissues, we consider the possibility that the fox cub was coinfected with the viral agent very unlikely. Similarly, inclusion bodies that can be seen in dogs with canine distemper virus were not observed in the lungs, brain, or epithelial cells of the organs in the present case. In addition, infection with a canine protoparvovirus that could be associated with pulmonary haemorrhages and jaundice could be excluded, because those specific lesions in the intestinal tract were also absent.

Taken together, we conclude that the young fox presented most probably died from a *Leptospira* spp. and *H. canis* coinfection associated with acute haemolytic anaemia and respiratory distress.

## 4. Conclusions

The present case reports a severe *Leptospira* spp. and *H. canis* coinfection in a juvenile red fox that is most likely associated with a fatal outcome, and thus, it represents the first case of a clinically manifested infection with these two pathogens described in this wild canid species worldwide. Based on our results, the pathogenic natures of the *Leptospira* spp. and *H. canis* in red foxes appear to be similar to those in infected dogs, and we believe that such fatal cases of infection may commonly occur in nature. However, further studies are required to identify other factors that may influence the severity and the pathogenesis of *Leptospira* spp. and/or *H. canis* infections in red foxes.

## Data Availability

All data generated or analysed during this study are included in the published article.
